# Identifying predictors of medication-related harm in older populations: a latent class analysis approach

**DOI:** 10.1093/ageing/afaf227

**Published:** 2025-08-21

**Authors:** Ross Brannigan, Juliane Frydenlund, David J Williams, Frank Moriarty, Emma Wallace, Ciara Kirke, Kathleen E Bennett, Caitriona Cahir

**Affiliations:** Royal College of Surgeons in Ireland, School of Population Health, Dublin, Leinster, Ireland; Royal College of Surgeons in Ireland, School of Population Health, Dublin, Leinster, Ireland; Department of Geriatric and Stroke Medicine, Royal College of Surgeons in Ireland, Dublin, Leinster, Ireland; Royal College of Surgeons in Ireland, School of Pharmacy and Biomolecular Sciences, Dublin, Ireland; Department of General Practice, University College Cork School of Medicine, Cork, Ireland; National Quality and Patient Safety Directorate at Health Service Executive, Dublin, Ireland; Royal College of Surgeons in Ireland, School of Population Health, Dublin, Leinster, Ireland; Royal College of Surgeons in Ireland, School of Population Health, Dublin, Leinster, Ireland

**Keywords:** medication-related harm, adverse drug reactions (ADR), latent class analysis (LCA), older patients, high risk drugs, quality of life, functional impairment, hospitalisation

## Abstract

**Background:**

The aim of this study was to apply latent class analysis to identify underlying groupings of predictors, including drug classes and clinical predictors, co-occurring in older people at higher risk of medication-related harm.

**Method:**

The Adverse Drug reactions in an Ageing PopulaTion cohort was used (*N* = 798 patients aged ≥65 years admitted acutely to hospital). Seven drug classes; antithrombotic agents, diuretics, renin-angiotensin-aldosterone system, calcium channel blockers, beta-blocking agents, psychoanaleptics, non-steroidal anti-inflammatory drugs, and comorbidity, frailty and significant polypharmacy (10+ different drug classes) were included as potential predictors of medication-related harm. Medication-related harm outcomes included adverse drug reactions (ADR)-related hospital admissions, health-related quality of life, functional impairment and emergency department visits. Determination of the best number of latent classes was based on standard comparison of fit statistics. Univariate and multivariable logistic, linear and Poisson regression models were used to examine the associations between the latent groups and the medication-related harm outcomes.

**Results:**

A five class model was determined to fit best; (i) high-risk prescribing and polypharmacy group (*N* = 245); (ii) low-risk group (*n* = 138); (iii) high-risk prescribing only group (*N* = 332); (iv) antihypertensive group (*N* = 18); and (v) psychoanaleptics and polypharmacy group (*N* = 65). Patients in both the high-risk prescribing and polypharmacy group (a.OR = 2.59, 95%CI = 1.51–4.44) and the high-risk prescribing only group (a.OR = 2.85, 95%CI = 1.57–5.20) were more likely to have an ADR-related hospital admission, with the high-risk prescribing and polypharmacy group also having statistically significant higher functional impairment (β = 1.21, 95% CI = 0.09, 2.33) compared to those in the low-risk group.

**Conclusion:**

Identifying distinct subgroups of older people based on their medications may lead to more targeted and tailored interventions to reduce potential medication-related harm.

## Key Points

- This is the first study to use LCA to identify groups of older people at higher risk of medication-related harm
- Five distinct groups of older patients, based on their prescribed medications and co-morbidities at hospital admission were determined
- Patients in the high-risk prescribing and polypharmacy group were more likely to have an ADR-related hospital admission and higher functional impairment at hospital admission
- Patients in the high-risk prescribing only group were more likely to have an ADR-related hospital admission

## Background

The World Health Organisation has identified Medication Safety as the theme of its third Global Patient Safety Challenge and its goal is to reduce severe, avoidable medication-related harm by 50% globally in the next 5 years [1]. While medicines play an essential role in the treatment of illness, managing chronic conditions, maintaining health and wellbeing and improving life expectancy, medication-related harm is also common and includes, adverse drug reactions (ADRs; noxious and unintended responses to medicinal products), adverse drug events (ADEs; a broader term for injuries related to medicine use) and other adverse health outcomes including hospitalisations, reduced health related quality of life (HRQOL), and functional impairment [2–4]. Older persons (aged ≥65 years) are at a greater risk of medication-related harm due to increased multimorbidity and medication utilisation and a variety of physiological changes affecting the pharmacokinetics and pharmacodynamics of medications [5]. Approximately 10% of hospital admissions in older populations are attributable to medication-related harm [6].

In order to reduce medication-related harm in older populations we need to develop methods that enable us to identify those older people who have an increased likelihood of experiencing medication-related harm. While a number of medications, including antiplatelets, anticoagulants, non-steroidal anti-inflammatory drugs (NSAIDs), and diuretics, have all been implicated in medication-related harm in older people, as well as increasing age, polypharmacy and multimorbidity, the predictive factors and the interrelationships between them are still poorly understood [7, 8]. Potentially inappropriate prescribing (PIP) and potential prescribing omissions (PPOs) criteria are highly prevalent in older people [9] and have been shown to contribute to between 7% and 17% of ADR-related hospital admissions [10] and to be associated with ADEs, hospitalisation and reduced HRQOL [11, 12]. However, while PIP/PPO indicators are informative, the lists of criteria are extensive and time consuming to apply in practice and are limited by their single drug/disease orientated approach [13].

Identifying older people at increased risk of medication-related harm is challenging due to the complexity of medication management in older people and the difficulty for healthcare providers and patients to identify potential medication-related harm and to differentiate these presentations from symptoms of chronic disease or frailty [14]. Given the challenges, it may be worthwhile considering other approaches/methodologies for identifying those older patients living with multiple long term health conditions who are most at risk of medication-related harm, compared to previous research [8]. Latent Class Analysis (LCA) is a statistical method used to identify subtypes of related cases or groups of people (latent classes) that are similar with respect to a set of observed characteristics. [15] LCA has been used in medical research, to determine the probability of disease based on several observed factors when the disease itself does not have a standard diagnostic measure [16]. However, its utilisation in the context of risk assessment for medication-related harm in older people is limited. LCA may be used to ascertain natural underlying groups of older people, based on their medications, morbidity and functional impairment, who may be more likely to experience medication-related harm. Such an approach may become a useful decision support tool for clinicians and could help prioritise medicines reconciliation and avoid prescribing cascades (where a drug is prescribed to manage the symptom caused by another) in those older people at highest risk [5]. The aim of this study was to apply LCA to identify underlying groupings of predictors, including drug classes and other clinical predictors, which co-occur in those older people who may be more likely to experience medication-related harm outcomes.

## Methods

### Data source

This study used the Adverse Drug reactions in an Ageing PopulaTion (ADAPT) cohort (*N* = 798), a cross-sectional study designed to examine the prevalence and predictors of ADR-related hospital in all patients aged ≥65 years admitted acutely to a large tertiary referral hospital in Ireland over an 8 month period (November 2016–June 2017). [17, 18] Within the cohort, there are 361 admissions with an ADR, and a further 437 individuals randomly selected as to serve as a baseline comparative group, all without ADR-related hospitalisations. The mean age of the ADAPT cohort (N = 798) was 80.85 (SD = 7.56), with 256 (32%) patients aged over 85 years and 417 (52%) who were female. In total, 324 (41%) patients had a Charlson Comorbidity Index score ≥ 3 313 (39.2%) experienced significant polypharmacy and 441 (55%) were determined to be frail as per the Triage Risk Screening Tool [9].

A subset of patients from this initial cross-sectional study were invited to complete a questionnaire of patient-reported health outcomes (HRQOL, functional impairment, health service use) at hospital admission (*N* = 350). A protocol and detailed description of the ADAPT cohort has been previously published [17–19].

### Potential predictors of medication-related harm

Six drug classes were identified, through their Anatomical Therapeutic Chemical (ATC) classification, as being associated with ADR-related hospital admissions in previous research using the ADAPT cohort and were included as potential predictors of medication-related harm in the LCA model [18]; antithrombotic agents (ATC: B01), diuretics (ATC: C03), agents acting on the renin-angiotensin-aldosterone system (RAAS) (ATC: C09), calcium channel blockers (ATC: C08), beta-blocking agents (ATC: C07), psychoanaleptics (ATC: N06). In addition we included NSAID (ATC: M01), comorbidity, frailty and significant polypharmacy (10+ different drug classes) as there is evidence of their association with ADR-related hospital admissions and medication-related harm in general as per previous systematic reviews [3, 6, 20]. Co-morbidity was measured using the Charlson co-morbidity index (≥3 points) [21]. Frailty was measured using the Triage Risk Screening, which has been shown to be predictive of emergency department (ED) revisits, hospitalisation and nursing home placement in older ED patients [22].

### Medication-related harm outcomes

The primary medication-related harm outcome was an ADR-related hospital admission and secondary outcomes included HRQOL, functional impairment and ED visits. Within the ADAPT cohort (*N* = 798), ADR-related admissions were determined on hospital admission using a multifaceted review of each admission to assess the likelihood of the ADR being a reason for admission (cause of admission or contributing to admission) in the context of the patient’s medication, medical diagnoses, medical history and investigations and using validated algorithms [23]. In total, 361 (11.7% 95% CI: 10.5%; 12.8%) patients were determined to have an ADR-related admission within the ADAPT cohort. A detailed analysis of the characteristics and the nature of the ADR-related hospital admissions (causality, severity and preventability) have been published previously [17, 18].

HRQOL, functional impairment and ED visits were measured using a patient questionnaire (*N* = 350). HRQOL was measured using the EQ-5D-5L score at hospital admission [24]. EQ-5D-5L health states were converted into a single utility value for each patient based on Irish population value set [25]. Groningen Frailty Index was used to measure functional impairment (loss of function and resources) at hospital admission and includes four domains: [1] physical (mobility functions, multiple health problems, physical fatigue, vision, hearing); [2] cognitive (cognitive dysfunction); [3] social (emotional isolation) and; [4] psychological (depressed mood and feelings of anxiety) [26]. The number of ED visits in the previous 3 months was measured at hospital admission per patient self-report.

### Covariates

Age, sex (male vs. female) and private health insurance status (yes/no) were included as covariates. Age and sex were measured at hospital admission for all (*N* = 798). Private health insurance status was only available for the subset of patients who completed the questionnaire of patient-reported health outcomes (*N* = 350).

### Data analysis

#### Determination of latent class groups

The latent class creation was carried out using the Penn State Stata plugin, DoLCA and the connected bootstrapping plugin. Variable selection for the LCA, based on the potential predictors of medication-related harm, was firstly determined using an all-inclusive model, then repeated with the exclusion of each individual variable, using the LCA entropy score as a guide to determine variable inclusion; as entropy is a measure of how well defined the groups are within the LCA [27]. Models with increasing numbers of latent classes were fit and determination of optimal class numbers was based on standard comparison of fit statistics, specifically the Akaike Information Criterion, Bayesian Information Criterion (BIC) and the adjusted BIC as well as input surrounding the clinical interpretation of classes. Bootstrap likelihood ratio tests were also used to compare individual classes.

After determining the number of latent classes, each participant had a probability score for placement into each class. Briefly a posterior probability is assigned to each individual, for each class, essentially conveying the likelihood they would be placed within the class. In using posterior probability, every member of the cohort has a probability of placement into each of the groups; however, the reported N for each of the classes is the number of people who have their highest probability of placement. Using this approach usually shows similar results to a deterministic placement approach (where every member is assigned to one group only), however, gives a greater margin of error, observed in wider confident intervals. Overall, this approach is more appropriate to exploratory work.

#### Association between latent class groups and medication-related harm (outcomes)

Univariate and multivariable logistic, linear and Poisson regression models were used to examine the associations between the latent groups and ADR-related hospital admissions (logistic), HRQOL (linear), functional impairment (linear) and number of ED visits (Poisson), respectively. All multivariable regression models were adjusted for age and sex. The models examining the associations between the latent groups and HRQOL, functional impairment and number of ED visits, were also adjusted for health insurance status. Univariate and multivariable linear regression models (HRQOL, functional impairment) used robust standard errors (sandwich estimator) to control for mild violation of the homoskedasticity and normality distribution assumption in linear regression [28]. The data was analysed using Stata Version 18.0 [29]. Missing data was managed using listwise deletion on an analysis-by-analysis basis.

## Results

### Determination of the latent class groups

**Figure 1:**
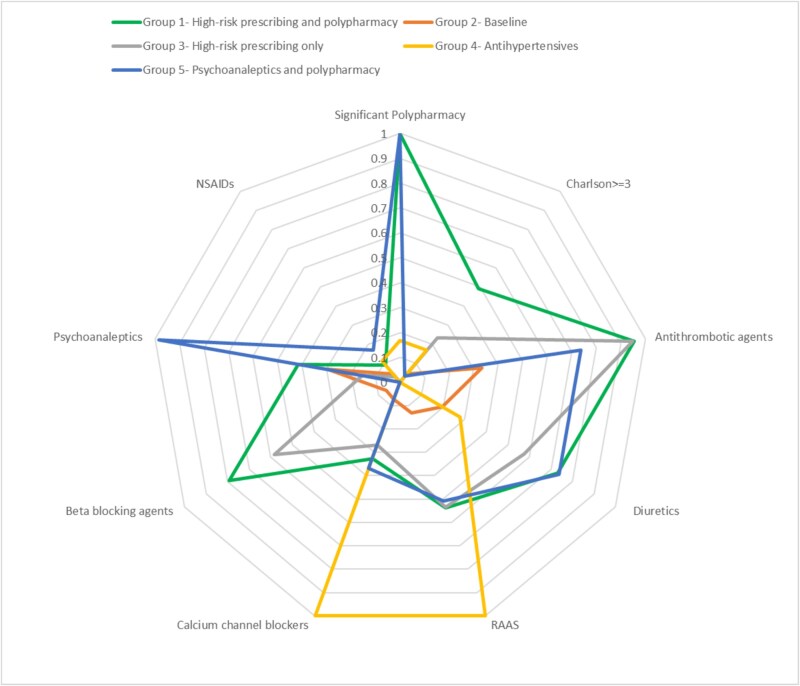
The five latent groups identified through LCA examining comorbidity, significant polypharmacy and the prescription of antithrombotic agents, diuretics, RAAS inhibitors, calcium channel blockers, beta blocking agent, psychoanaleptics and NSAIDs. Values indicate the probability of said characteristic occurring in each group.

We found that a model inclusive of comorbidity (≥3 points), significant polypharmacy and the prescription of antithrombotic agents (ATC: B01), diuretics (ATC: C03), RAAS inhibitors (ATC: C09), calcium channel blockers (ATC: C08), beta blocking agents (ATC: C07), psychoanaleptics (ATC: N06), NSAIDs (ATC: M01), and exclusive of frailty scores was the best fit. Model selection identified both a 4-class model and a 5-class model. The bootstrap likelihood ratio test was used to compare these two class models, demonstrating a significant difference (*P* = 0.006), suggesting the 4-class model may be too restrictive, or there was otherwise a benefit in using a 5-class model over a 4-class model. Comparing the 5-class model to a 6-class model, the bootstrap likelihood ratio test was insignificant, suggesting the 6-class model showed no benefit over the 5-class model. Therefore, a 5-class model was determined to be the best fit to the data (Figure 1). The fit statistics for this model as well as the bootstrapping analysis can all be seen in Supplementary Table 1.

### Description and baseline characteristics of each latent class group

**Figure 2:**
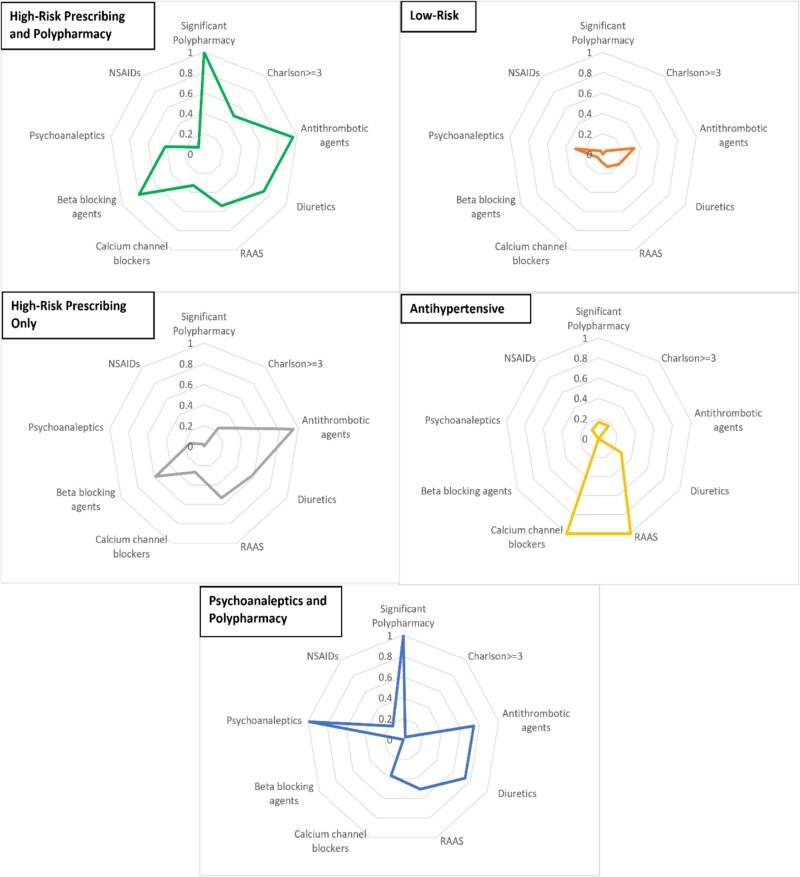
The characteristics of each of the five individual latent class groups. Values indicate the probability of said characteristic occurring in each group. Renin-angiotensin-aldosterone system (RAAS); Non-steroidal anti-inflammatory drugs (NSAIDs).

The general descriptions and characteristics of each latent group are presented in Figure 2. The first group (Group 1; Figure 2) had 245 (30.70%) participants, with 144 females and a mean age of 81.32 years (SD = 7.39), and was characterised by a high proportion of significant polypharmacy, a high proportion of use of antithrombotic agents, diuretics and beta-blocking agents, as well as a moderate use of RAAS inhibitors, calcium channel blockers and psychoanaleptics. This group was labelled a high-risk prescribing and polypharmacy group.

The second group (Group 2; Figure 2) had 138 (17.29%) participants, 80 females, mean age 79.86 years (SD = 7.51), and was characterised by a low proportion of all predictors and was labelled a low-risk group and was the baseline group for all analyses.

The third group (Group 3; Figure 2) was the largest group and had 332 (41.60%) participants, 132 females, with a mean age of 80.83 (SD = 7.52), and was characterised by similar drug class usage to Group 1, including a high use of antithrombotic agents, and similar but reduced use of diuretics, RAAS inhibitors and beta-blocking agents. However, this group had none or a very low proportionate use of psychoanaleptics, NSAIDS, significant polypharmacy and co-morbidity. Group 3 was labelled a high-risk prescribing only group.

The fourth group (Group 4; Figure 2) was the smallest group with only 18 (2.26%) participants, with 11 females and a mean age of 81.39 years (SD = 11.26), and was characterised by high proportionate use of RAAS inhibitors and calcium channel blockers, but low levels of all the other predictors and was labelled the antihypertensive group.

The last group (Group 5; Figure 2) had 65 (8.15%) older participants, with 50 females and a mean age of 81.18 years (SD = 7.26) and was characterised by a high proportion of significant polypharmacy and use of psychoanaleptics, and a moderate proportion of antithrombotic agents, diuretics, and RAAS inhibitors, calcium channel blockers and was labelled psychoanaleptics and polypharmacy group.

### Association between latent class groups and medication-related harm outcomes

For the primary outcome, those in the high-risk prescribing and significant polypharmacy group (Group 1 AOR 2.59 95% CI:1.51–4.44) or the high-risk prescribing only group (Group 3 AOR 2.85 95% CI:1.57–5.20) were almost three times more likely to have an ADR-related hospital admission compared to those in the low-risk group (Group 2) after adjusting for age and sex (Table 1).

**Table 1:** Unadjusted and adjusted odds ratios (AOR) and 95% CI for ADR-related hospital admissions by group placement (N = 798)

Group	ADR-related hospital admission (N %)	Non-ADR hospital admission **(N %)**	Unadjusted OR (95% CI)	Adjusted OR (95%CI)
Group 1- High-risk prescribing and polypharmacy	122 (15.29)	123 (15.41)	2.71 (1.59, 4.61)	2.59 (1.51–4.44)
Group 2- Low Risk	44 (5.51)	94 (11.78)	Ref.	Ref.
Group 3- High-risk prescribing only	160 (20.05)	172 (21.55)	2.74 (1.52, 4.94)	2.85 (1.57–5.20)
Group 4- Antihypertensives	7 (0.88)	11 (1.38)	2.07 (0.70, 6.09)	2.29 (0.76–6.95)
Group 5- Psychoanaleptics and polypharmacy	28 (3.51)	37 (4.64)	1.83 (0.8, 4.02)	1.65 (0.74–3.68)

^a^adjusted for age, sex and frailty status (Not Frail/Frail).

For secondary outcomes, those in the high-risk prescribing and significant polypharmacy group also had statistically significant higher functional impairment (Group 1 β = 1.21 95% CI 0.09, 2.33) compared to the low-risk group (Group 2) at hospital admission after adjusting for age, sex, the presence of health insurance and frailty (Table 2). Patients in the antihypertensive group (Group 4) had a statistically significant higher reported HRQOL (Table 3) and lower functional impairment (Table 3) than the low-risk group (Group 2) at hospital admission, though only six participants were in Group 4. There were no significant differences in the number of ED visits in the previous 3 months between the latent class groups (Supplementary Table 2).

**Table 2:** Unadjusted and adjusted coefficients and 95% CI for functional impairment by group placement (*N* = 314)

Group	N (%)	Median score for functional impairment (IQR)	Unadjusted coefficient (95% CI)	Adjusted coefficient (95%CI)
Group 1- High-risk prescribing and polypharmacy	108	7 (5, 10)	1.78 (0.58, 2.97)	1.21 (0.09, 2.33)
Group 2- Low Risk	51	5 (3, 7)	Ref.	Ref.
Group 3- High-risk prescribing only	127	5 (3, 6)	−0.79 (−2.08, 0.51)	−0.71 (−1.90, 0.49)
Group 4- Antihypertensives	6	3 (3, 4)	−2.67 (−4.13, −1.21)	−2.07 (−3.49, 0.65)
Group 5- Psychoanaleptics and polypharmacy	22	7.5 (5.5, 11.5)	2.47 (0.47, 4.48)	1.90 (−0.08, 3.88)

^a^adjusted for age, sex, frailty status and presence of health insurance, using robust standard errors. Data were missing for 36 patients. Confidence Interval (CI); Inter-quartile range (IQR).

**Table 3:** Unadjusted and adjusted coefficients and 95% CI for health related quality of life (HRQOL) by group placement (N = 324)

Group	N (%)	Median score for HRQOL (IQR)	Unadjusted coefficient (95% CI)	Adjusted coefficient (95%CI)
Group 1- High-risk prescribing and polypharmacy	110	0.51 (0.20,0.74)	−0.08 (−0.23, 0.08)	−0.7 (−0.23, 0.08)
Group 2- Low Risk	52	0.63 (0.17,0.91)	Ref.	Ref.
Group 3- High-risk prescribing only	133	0.73 (0.45, 0.92)	0.14 (−0.02, 0.30)	0.13 (−0.03, 0.29)
Group 4- Antihypertensives	6	0.85 (0.68, 1)	0.45 (0.25, 0.65)	0.46 (0.29, 0.63)
Group 5- Psychoanaleptics and polypharmacy	23	0.41 (0.11, 0.70)	−0.22 (−0.50, 0.05)	−0.24 (−0.52, 0.05)

^a^adjusted for age, sex and presence of health insurance, using robust standard errors. Data were missing for 25 patients. Confidence Interval (CI); Inter-quartile range (IQR).

## Discussion

Through the application of LCA, we classified older patients, based on their prescribed medications and co-morbidities (≥3 points), at hospital admission, into five distinct groups representing different categories of older patients at risk of medication-related harm. Group 1 (high-risk prescribing group and polypharmacy) and Group 3 (high-risk prescribing only) were both characterised by a high use of antithrombotic agents and the use of diuretics, RAAS inhibitors and beta-blocking agents, as well as having the highest scores in both polypharmacy and comorbidities (Charlson). Patients in both groups were significantly more likely to have an-ADR related hospital admission. Significant polypharmacy was highly characteristic of Group 1 (high-risk prescribing group and polypharmacy) and Group 5 (psychoanaleptics and polypharmacy) only and patients in Group 1 were significantly frailer on hospital admission. Additionally, Group 5 (psychoanaleptics and polypharmacy). None of the groups were characterised by the use of NSAIDs..

This is one of few studies to use LCA to identify groups of older people at higher risk of medication-related harm. LCA has also being used to develop multidimensional measures of polypharmacy with varying pharmacotherapeutic and clinical appropriateness, rather than standard cut-offs based on numbers of medications and the latent groups have been found to be associated with 3-year mortality and institutionalisation. [30, 31] Given the heterogenous nature of older people living with multimorbidity, LCA provides a method to potentially identify clusters or groups of older people, who may have an increased risk of medication-related harm, based on combinations of risk predictors, rather than focusing on individual patient attributes [14, 32]. A UK study, using LCA, identified five different clusters of conditions in people with multiple long-term conditions and found these clusters to differ in their association with mortality, hospitalisations and general practice use [33]. In the USA, LCA has been used to identify four classes of homebound older individuals differentiated by their health, function, sociodemographic characteristics, and caregiving context, with differing risk of one-year mortality [34].

LCA offers a complementary approach to existing methods of identifying factors related to medication-related harm. To date current ADR and ADE risk prediction regression models in older populations have been determined to have relatively modest performance in external validation and none have been implemented into clinical practice [8]. A newly developed risk prediction model (ADAPTiP) using the current dataset, consists of similar drug classes (antithrombotic agents, diuretics, RAAS agents) as commonly used by Groups 1 and 3, as well as four primary presenting complaints (bleeding, gastrointestinal and respiratory disorders and syncope), increasing age and chronic lung disease [35]. While the LCA model is not a formal risk prediction tool, both ADAPTiP and the LCA-derived classification approach require external validation in independent cohorts, ideally across diverse healthcare settings, to strengthen the evidence base and support potential clinical application [36, 37].

### Limitations

This study has a number of limitations. The study cohort included older people living with multimorbidity prescribed a large number of medications, who were screened for an ADR on admission to hospital and the findings may not be generalisable to other older populations or across other healthcare settings. The determination of an ADR-related hospital admission included a multifaceted review of each suspected ADR incorporating clinical judgement, medical record review, and the application of a number of validated algorithms, but there is still a potential risk of misclassification. While a gold-standard medication reconciliation list was completed for each patient, it is unknown if patients were actually taking their medication as prescribed. Due to the cross-sectional nature of the study, causal and temporal associations between the latent class groups and health outcomes cannot be inferred and there is always the potential for residual confounding. Self-reported health outcomes (HRQoL, functional impairment, ED visits) were available only for a subset of the cohort. Although family members or caregivers could respond on behalf of participants, individuals who were very unwell may have been systematically excluded, potentially introducing bias. The association between the different categories of older patients at risk of medication-related harm and health outcomes (HRQOL, functional impairment and ED visits) requires further investigation in larger population cohorts It is worth noting that for the secondary analyses, the inclusion of the Charlson within the LCA may lead to potential confounding of the results dues to potentially high impacts of ED, HRQOL and functional impairments.

### Implications

Risk stratification of older people living with multimorbidity prescribed multiple medications may have important implications for clinical practice. It enables healthcare professionals to identify those groups of older individuals who would benefit most from structured medication reviews, deprescribing, therapeutic monitoring and pharmacotherapeutic adjustments; interventions known to reduce the risk of medication-related harm [38]. Antithrombotics, diuretics and RAAS inhibitors have all been implicated in ADR-related hospital admissions and have a high innate toxicity in older populations [35, 39]. The likelihood of experiencing medication-related harm has also been shown to increase significantly with significant polypharmacy (10+ different drug classes) and polypharmacy has also being identified as a major contributor to the development of frailty [40, 41]. Narrowing the focus to those on particular high-risk drug classes or drug class combinations may offer a pragmatic approach to interventions aiming to reduce medication-related harm in a healthcare system with ever-increasing demands on its resources and in an ever-increasing older population. Annual structured medication reviews and reducing the number of high-risk medications in older populations have been shown to prevent ADRs and revert or delay frailty [40, 41].

## Conclusion

This study found that combinations of certain high-risk drug classes in older populations have a distinct association with medication-related harm, including ADR-related hospital admissions and functional impairment. Identifying distinct subgroups of older people based on their medications may lead to more targeted and tailored interventions to reduce potential medication-related harm and better allocation of healthcare resources.

## Supplementary Material

aa-25-0841-File002_afaf227
