# Supplementary material for: Identifying predictors of medication-related harm in older populations: a latent class analysis approach
Source: Age Ageing. 2025 Aug 21;54(8):afaf227. doi: 10.1093/ageing/afaf227 (PMC12368848; doi:10.1093/ageing/afaf227)
Supplement: aa-25-0841-File002_afaf227 [file aa-25-0841-file002_afaf227.docx]

**Supplementary 1**

**Entropy Comparison**

Given the subjective nature of finding the best combination of variables, we used entropy as a guide for variable inclusion, using a stepwise exclusion method, running models removing one variable at a time. Two models reached a consistent entropy value of above 0.70, specifically models between the 2-class model and a 5-class model, , the first model excluding frailty, the second excluding diuretic drugs. In this instance, as fit statistics cannot be compared across two models, we decided based on individual odd ratio associations with ARD-related hospital admissions, our primary outcome. Being frail produced an odds ratio of 1.43 (95% CI= 1.08 – 1.90), while being prescribed diuretics produced an odds ratio of 1.56 (95% CI = 1.17 – 2.07), indicating that including diuretics may be more valuable an inclusion at identifying risk groups.

**Model Selection**

Once we decided on our included variables, we had to select our model. Literature states that the BIC is the best statistic to base model selection off (Chen et al., 2017), however the AIC can also provide insight. Fit statistics showed us that there were 2 potential options, with the AIC, adjusted BICs (see fit statistics in Supplementary Table 1) being virtually the same, these being the 4 and 5 class models, and the BIC also being close. In this scenario, where these is no clear standout optimal class, we can use a bootstrapping method to further reinforce our choice. Bootstrapping compares K (4) and K+1 (5) classes, and determines whether K (4) classes could be too restrictive. Our results indicated that 4 classes was too restrictive for our model (significant bootstrapping results). Additionally, we also compared K (5) and K+1 (6) classes to see if 5 classes adequately represented our data and found that it did (non-significant bootstrapping result). This led us to our final selection of a five-class model.

**Supplementary Table 1: Fit statistics for 4 and 5 class models**

|  | 2 class | 3 class | 4 class | 5 class | 6-class |
| --- | --- | --- | --- | --- | --- |
| df | 492 | 482 | 472 | 462 | 452 |
| EntropyRsqd | .8549 | .7060 | .7496 | .7817 | .6865 |
| EntropyRaw | 80.2693 | 257.7362 | 277.0580 | 280.3234 | 448.3210 |
| AdjustedBIC | 564.4136 | 540.5936 | 517.3970 | 519.1075 | 546.5180 |
| caic | 643.7491 | 661.6846 | 680.2435 | 723.7094 | 792.8755 |
| bic | 624.7491 | 632.6846 | 641.2435 | 674.7094 | 733.8755 |
| aic | 535.7890 | 496.9034 | 458.6412 | 445.2861 | 457.6311 |
| Gsquared | 497.7890 | 438.9034 | 380.6412 | 347.2861 | 339.6311 |
| loglikelihood | 4050.1731 | -4020.7303 | -3991.5992 | -3974.9217 | -3971.0942 |
| iteration | 229 | 307 | 462 | 1137 | 1457 |
| Bootstrapping Results | 0.002* | 0.002* | 0.006* | 1 | 0.002* |

Chen, Q., Luo, W., Palardy, G.J., Glaman, R., McEnturff, A., 2017. The Efficacy of Common Fit Indices for Enumerating Classes in Growth Mixture Models When Nested Data Structure Is Ignored: A Monte Carlo Study. SAGE Open 7(1), 2158244017700459. <https://doi.org/10.1177/2158244017700459>

**Supplementary Table 2**

**Supplementary Table 2. Unadjusted and adjusted incidence-rate ratios (IRR) and 95% CI for number of ED visits by group placement (N=324)**

| Group | N (%) | Median number of ED visits (IQR) | Unadjusted IRR (95% CI) | Adjusted IRR (95%CI) |
| --- | --- | --- | --- | --- |
| Group 1- High-risk prescribing and polypharmacy | 110 | 1 (0,2) | 1.12 (0.77, 1.62) | 1,09 (0.75 - 1.60) |
| Group 2- Low Risk | 52 | 1 (0,1) | Ref. | Ref. |
| Group 3- High-risk prescribing only | 133 | 1 (0,1) | 0.93 (0.61, 1.40) | 0.88 (0.57 - 1.37) |
| Group 4- Antihypertensives | 6 | 0 (0,1) | 0.27 (0.07, 1.11) | 0.30 (0.06 - 1.39) |
| Group 5- Psychoanaleptics and polypharmacy | 23 | 1 (0,1) | 0.96 (0.53, 1.74) | 1.07 (0.58 - 1.99) |

*adjusted for age and sex and presence of Health Insurance. Data was missing for 25 patients

**Supplementary Table 3**

Supplementary Table 3. Probability value for each classifying variable by individual class.

| Class | Significant Polypharmacy | Charlson>=3 | Antithrombotic agents | Diuretics | RAAS | Calcium channel blockers | Beta blocking agents | Psychoanaleptics | NSAIDs |
| --- | --- | --- | --- | --- | --- | --- | --- | --- | --- |
| Group 1- High-risk prescribing and polypharmacy | 1 | 0.49 | 0.96 | 0.73 | 0.54 | 0.33 | 0.79 | 0.42 | 0.09 |
| Group 2- Baseline | 0 | 0.04 | 0.33 | 0.20 | 0.13 | 0.07 | 0.07 | 0.30 | 0.04 |
| Group 3- High-risk prescribing only | 0 | 0.23 | 0.95 | 0.58 | 0.54 | 0.27 | 0.58 | 0.15 | 0.03 |
| Group 4- Antihypertensives | 0.1667 | 0.17 | 0 | 0.28 | 1 | 1 | 0 | 0 | 0.11 |
| Group 5- Psychoanaleptics and polypharmacy | 1 | 0.03 | 0.74 | 0.74 | 0.51 | 0.37 | 0 | 0.98 | 0.17 |
